# 

**Published:** 1915-11-06

**Authors:** 


### PASSING EVENTS AND TOPICS.

### The Badge for the Unfit.

Considerable difference of opinion has been expressed regarding the wearing of the proposed khaki armlet by those men who are exempt from Army service by reason of medical unfitness. If there is to be a distinguishing mark on those armlets worn by the unfit, as is stated to be the case, there may be some solid excuse for objections. It will not be a matter for surprise if some of those "entitled" to this badge hesitate to wear it.

### British Hospitals Association.

The annual meeting of this Association will be held at the Westminster Hospital, London, on the 18th inst., at 3 p.m., Mr. H. Wade Deacon, chairman of the Royal Infirmary, Liverpool, in the chair. At the conclusion of the formal business Mr. Sydney A. Smith, F.S.I., will read a paper on "Provisional Valuations under Finance (1909-10) Act, 1910, as they affect the Voluntary Hospitals." If a representative attendance is desired it would be well for Mr. Smith to indicate in advance his main points, and when and how they touch the voluntary hospitals.

### Old Bandages Made New.

The use of lint has been forbidden in Germany because the material from which it is made can be used to good advantage for other purposes. There is no need for any great exercise of the imagination to arrive at a conclusion as to what these "other purposes" are. It appears, however, from information obtained through a neutral source, that there is no likelihood of any shortage of bandage material in Germany, one reason being that old bandages, after thorough disinfection, are being used again.

### Russian Resistance to Typhus.

In a recent letter the Berlin correspondent of the official organ of the American Medical Association states that it has been determined that the Russians display a much greater resistance to typhus than the Germans. "Whereas," he writes, "the mortality among the Russians is only 2 per cent., the mortality among the German doctors and nurses is about — per cent." Unfortunately the percentage in the latter cases was deleted from the original letter by the Censor, but the definite statement regarding the Russian resistance to typhus is interesting.

### Alleged Personation of a Doctor.

One of those cases of personation or alleged personation of a registered medical practitioner which from time to time are brought to light was heard at the North London Police Court on October 30. The accused was Henry John Herring, and the evidence was to the effect that, falsely representing himself to be Dr. Edward Kerr Herring, of West Maitland, New South Wales, he obtained the position of *locum tenens* for Dr. Deighan, of Clapton Square, London, and later for another practitioner at Cambridge. He pleaded guilty to the charge of giving a false certificate of death, and was committed for trial at the Central Criminal Court.

### "Our Day" in Greater London.

The result of the collections in Greater London on October 21 was the handsome sum of £32,734 14s. 4d., this being the amount actually collected in the streets of London. It does not include large donations sent to the Joint Committee of the Red Cross and the Order of St. John. Westminster heads the list with £5,000, "The Baltic" coming next with £3,500. Of the suburbs Islington did best with a collection of £1,360, which record was closely followed by Wandsworth with a total of £1,300. When one remembers what an inclement day the "21st" was one can only wonder at the pluck of the ladies who collected this large total, mostly, no doubt, in coppers.

### A Seventy-Fifth Anniversary.

The Queen's Hospital, Birmingham, has been commemorating its seventy-fifth anniversary. The foundation-stone was laid in June 1840, and the wards were opened for the reception of patients on October 24, 1841. At the anniversary service the Lord Bishop of Birmingham (Dr. Russell Wakefield) referred in eloquent terms to the work accomplished by hospitals, especially at the present time. He afterwards made a tour of all the wards and much cheered the patients by his kindly words. The occasion was also taken to place in the chapel a mural tablet to commemorate the long and faithful services rendered to the hospital by its former superintendent, the late Mr. Arthur Richard Hume.

### Menthol Water for the Troops.

A report is current that a recent advance in the price of menthol has been occasioned by a large demand for the drug for an unexpected purpose—namely, the preparation of a warming drink for our troops in the trenches. While this is by no means improbable, one would surely think that oil of peppermint would be more suitable than menthol for this purpose. True, a saturated solution of menthol in water is sometimes used in medicine as a carminative and antispasmodic in place of peppermint water, but menthol is more liable to impair the digestion than is peppermint oil. It would be interesting to know whether such a beverage was used in the Russo-Japanese War, and whether the idea, like the menthol, comes from Japan.

### German Precautions Against Epidemics.

Some idea of the strenuous efforts that are being made by the German military authorities to suppress disease can be obtained from the following extract from the Berlin letter to the *Journal of the American Medical Association*: "The entire Eastern front has been converted into a huge filter to guard against the spread of epidemics and the carrying of disease. Each man in the army, from the General down to the private, prisoners as well as our own troops, must conform to the regulations made by the military and sanitary authorities before he can enter the German Empire." Disinfection stations are being built everywhere along the Eastern border, each of which consists of eight separate buildings in which 500 men can be treated at one time. Each building is divided into a "clean" and an "unclean" part, so that the mingling of people is impossible. At the entrance on the "unclean" side every man receives a white net in which he can place his clothing, and a brown net in which are placed his helmet, boots, leggings, and anything else that can be sterilised by dry heat. When the soldier is stripped of all his possessions he has a distinguishing mark placed round his neck corresponding to the number on his nets. He then has a bath, and after being thoroughly cleansed he is given new underclothing and passes on to the "clean" side of the building, where he receives his disinfected clothing. Surely a lesson in thoroughness.

### Buildings Contemplated.

AYTON.—At a general meeting of the East District Committee of the Berwickshire County Council, held at Ayton, it was agreed to consider the provision of a small-pox hospital for the East District at the next meeting of the committee.

CAMBRIDGE HEATH.—The Bethnal Green Board of Guardians invite tenders for the building of a phthisical shelter at the Military Hospital, Cambridge Heath, N.E. Forms of tender and all particulars may be obtained of Mr. D. Thomas, Clerk to the Guardians.

EDMONTON.—The Edmonton Guardians invite tenders for the erection of two temporary wards at the Edmonton Military Hospital. Particulars may be obtained from the architect, Mr. J. C. S. Mummery, 13 Fitzroy Square, W.

FAIRLEE.—The new cubic block built in connection with the Isle of Wight Joint Isolation Hospital at Fairlee, Newport, has been formally opened. Mr. Cocks was the architect and Mr. Williams the contractor.

GLASS HOUGHTON.—The Normanton and District Joint Isolation Hospital Committee have decided to ask the West Riding County Council for authority to provide small-pox accommodation at their own hospital instead of joining in a combined scheme. The approximate cost of the alterations is £900.

MAGHULL.—The Maghull Epileptic Homes Committee has decided to make a further addition to the colony at Maghull, and plans have been prepared accordingly. The new building is to be erected next to the Henry Cox Home, off Smithy Lane, Maghull, at a cost estimated at £15,000.

PONTARDULAIS.—A freehold site for a cottage hospital to serve the district of Pontardulais has been offered by Miss Roberts, of Llandremorfawr.

SPALDING.—The Local Government Board have declined to sanction a loan for the proposed extension of the nurses' quarters. The Guardians have decided to notify the Board that they propose to proceed with the work out of current income, as it is a necessity.

WARRINGTON.—The Council has approved plans for a small-pox hospital to be built at a cost of £1,800.

Rates of Subscription: Twelve months, 6/6; Six months, 4/-; Three months, 2/-, post free to any address in the United Kingdom. Abroad, Twelve months, 8/8; Six months, 5/-; Three months, 2/6, post free.—Address The Subscription Dept., "The Hospital," The Hospital Building, 28/29 Southampton Street, Strand, London, W.C.

